# Novel combinatorial approach: Harnessing HIV protease inhibitors to enhance amphotericin B’s antifungal efficacy in cryptococcosis

**DOI:** 10.1371/journal.pone.0308216

**Published:** 2024-08-01

**Authors:** Nour M. Alkashef, Mohamed N. Seleem

**Affiliations:** 1 Department of Biomedical Sciences and Pathobiology, Virginia-Maryland College of Veterinary Medicine, Virginia Polytechnic Institute and State University, Blacksburg, Virginia, United States of America; 2 Center for One Health Research, Virginia Polytechnic Institute and State University, Blacksburg, Virginia, United States of America; 3 Department of Microbiology and Immunology, Faculty of Pharmacy, Zagazig University, Zagazig, Alsharkia, Egypt; Gulu University, UGANDA

## Abstract

Cryptococcosis is a fungal infection that is becoming increasingly prevalent worldwide, particularly among individuals with compromised immune systems, such as HIV patients. Amphotericin B (AmB) is the first-line treatment mainly combined with flucytosine. The scarcity and the prohibitive cost of this regimen urge the use of fluconazole as an alternative, leading to increased rates of treatment failure and relapses. Therefore, there is a critical need for efficient and cost-effective therapy to enhance the efficacy of AmB. In this study, we evaluated the efficacy of the HIV protease inhibitors (PIs) to synergize the activity of AmB in the treatment of cryptococcosis. Five PIs (ritonavir, atazanavir, saquinavir, lopinavir, and nelfinavir) were found to synergistically potentiate the killing activity of AmB against *Cryptococcus* strains with ƩFICI ranging between 0.09 and 0.5 against 20 clinical isolates. This synergistic activity was further confirmed in a time-kill assay, where different AmB/PIs combinations exhibited fungicidal activity within 24 hrs. Additionally, PIs in combination with AmB exhibited an extended post-antifungal effect on treated cryptococcal cells for approximately 10 hrs compared to 4 hours with AmB alone. This promising activity against cryptococcal cells did not exhibit increased cytotoxicity towards treated kidney cells, ruling out the risk of drug combination-induced nephrotoxicity. Finally, we evaluated the efficacy of AmB/PIs combinations in the *Caenorhabditis elegans* model of cryptococcosis, where these combinations significantly reduced the fungal burden of the treated nematodes by approximately 2.44 Log10 CFU (92.4%) compared to the untreated worms and 1.40 Log10 ((39.4%) compared to AmB alone. The cost-effectiveness and accessibility of PIs in resource-limited geographical areas compared to other antifungal agents, such as flucytosine, make them an appealing choice for combination therapy.

## Introduction

Cryptococcosis is an opportunistic fungal infection caused primarily by the two closely related basidiomycetous species, *Cryptococcus neoformans* and *Cryptococcus gattii* [1]. This infection starts with inhalation of the spores or the desiccated yeast cells, which can colonize the entire respiratory tract and present with pneumonia-like symptoms [2]. In immunocompromised patients, the infection can progress to more severe forms, such as cryptococcal meningitis (CM), characterized by symptoms including fever, lethargy, headache, and photophobia to life-threatening increased intracranial pressure [3]. CM is one of the most common morbidities among immunocompromised individuals, particularly HIV patients [4]. In 2020, CM accounted for approximately 152,000 cases, resulting in 112,000 deaths, with a mortality rate up to 74.6%. Furthermore, 19% of HIV-related mortality was a consequence of this opportunistic infection [5].

Current therapeutic guidelines include three antifungals for CM treatment. Amphotericin B (AmB) is typically combined with flucytosine (5-FC) in the induction stage to ensure rapid clearance of the fungal burden within the cerebrospinal fluid (CSF). Fluconazole (FLC) is then used in moderate to low doses during consolidation and maintenance stages to ensure CSF sterilization [6]. However, limited access to 5-FC, mainly due to cost constraints, impedes treatment availability especially in high-burden areas such as Africa, South America, and Asia [7, 8]. In addition, the use of AmB with 5-FC requires careful monitoring due to concomitant toxicity such as nephrotoxicity, bone marrow depression, and hepatotoxicity [9]. Furthermore, the less toxic lipid-associated formulations of AmB are prohibitively expensive and scarce in resource-limited areas [10, 11]. In these regions, FLC is alternatively used at a high dose (1200 mg/kg) during the induction stage. However, this regimen is frequently associated with treatment failure and relapses due to its fungistatic nature [12].

**Fig 1:**
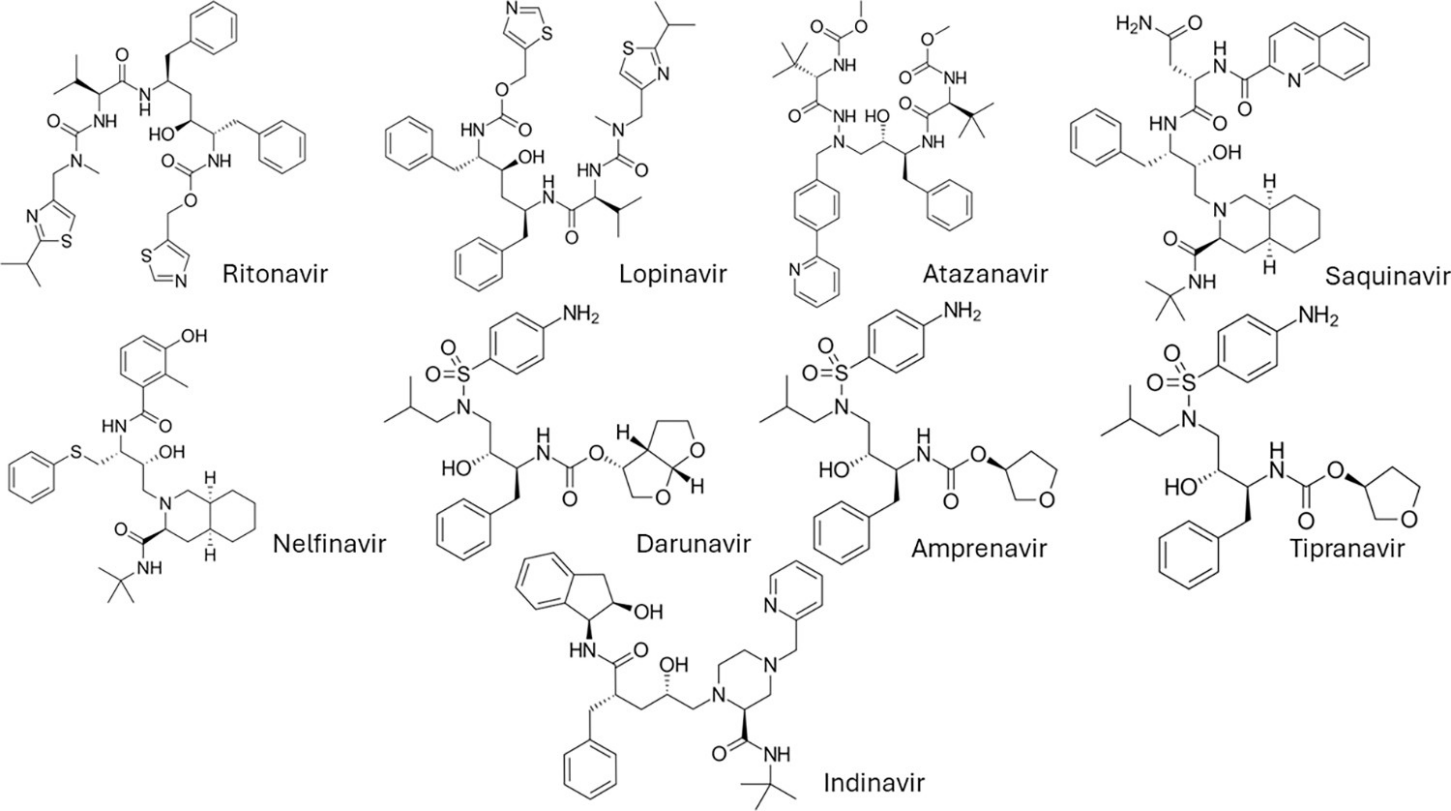
Structures of the PIs involved in the study.

HIV-protease inhibitors (PIs) are a cornerstone in the current regimen of highly active antiretroviral therapy. PIs primarily disable the activity of HIV aspartyl protease enzyme cleaving the immature polyprotein into mature proteins necessary for viral replication [13]. The introduction of antiretroviral therapy (ART) has demonstrated a significant improvement in the survival rate among HIV-infected patients with cryptococcosis [14]. Previously, our group reported the efficacy of PIs in enhancing the activity of azoles in vitro and in vivo by compromising the efflux system in emerging multidrug-resistant *Candida auris* [15–19]. In this study, we broadened our investigation to determine which of the nine FDA-approved PIs (Fig 1) has the potential to be utilized as adjuvants with AmB in the treatment of cryptococcosis. The ability of PIs to potentiate AmB activity was confirmed using a panel of in vitro techniques including standard microdilution checkerboard assays, time-kill assays, and post-antifungal effect (PAFE) assays. Additionally, the safety profile of this combination was evaluated on mammalian kidney cells to rule out the risk of increased nephrotoxicity. Finally, the in vivo efficiency of AmB/PIs was evaluated in the *Caenorhabditis elegans* nematode model of cryptococcosis.

## Material and methods

### Fungal strains, reagents, and chemicals

All fungal strains involved in this study are listed in Table 1. RPMI 1640 powder (with glutamine) and phenazine methosulfate (PMS) were purchased from Thermo Fisher Scientific (Waltham, MA). Yeast-peptone-dextrose (YPD) broth and YPD agar were obtained from Becton, Dickinson, and Company (Franklin Lakes, NJ). 3-(*N*-Morpholino) propanesulfonic acid (MOPS) was obtained from Sigma-Aldrich (St. Louis, MO). HIV-protease inhibitors were purchased from Ambeed (Arlington, IL). Ampicillin was obtained from Fisher Scientific (Pittsburgh, PA). 3-(4,5-dimethylthiazol-2-yl)-5-(3-carboxymethoxyphenyl)-2-(4-sulfophenyl)-2H-tetrazolium was purchased from Abcam (Waltham, MA). Kanamycin sulfate, streptomycin sulfate, and AmB were purchased from Chem-Impex International Inc. (Wood Dale, IL).

**Table 1:** Description of the isolates used in the study and their antifungal activity of amphotericin B and PIs.

Strain	Description	MIC (μg/mL)^a^					
		AmB	RTV	LPV	ATV	SQV	NLF
*C*. *neoformans* NR-50332	Isolated from human cerebrospinal fluid in San Gabriel, California in 1969, exhibiting low level of heteroresistance to fluconazole	0.5	>128	>128	>128	>128	>128
*C*. *neoformans* NR-50333	Isolated from human cerebrospinal fluid in Baltimore, Maryland in 1970, exhibiting high level of heteroresistance to fluconazole	1	>128	>128	>128	>128	>128
*C*. *neoformans* NR-50335	Derived from strain NIH 9, originally isolated in 1963 in Sassamanville, Pennsylvania, US. exhibiting high level of heteroresistance to fluconazole	1	>128	>128	>128	>128	>128
*C*. *neoformans* H99 ATCC-208821	Isolated from a patient with Hodgkin’s disease, NY, USA.	0.25	>128	>128	>128	>128	>128
*C*. *neoformans* NR-48773	Derived from strain H99O with a decreased virulence and mating ability.	1	>128	>128	>128	>128	>128
*C*. *neoformans* NR-48768	Derived from strain H99O with an increased virulence and mating ability	1	>128	>128	>128	>128	>128
*C*. *neoformans* NR-48774	Derived from strain H99O with decreased virulence and mating ability.	1	>128	>128	>128	>128	>128
*C*. *neoformans* NR-48770	Derived from strain H99O with increased virulence and mating ability	1	>128	>128	>128	>128	>128
*C*. *neoformans *NR-48771	Derived from strain H99O with decreased virulence and mating ability.	1	>128	>128	>128	>128	>128
*C*. *neoformans* NR-48772	Derived from strain H99O with an increased virulence and mating ability	1	>128	>128	>128	>128	>128
*C*. *neoformans* NR-48775	Derived from strain H99O with decreased virulence and mating ability.	1	>128	>128	>128	>128	>128
*C*. *neoformans var*. *grubii* NR-48776	Derived from strain H99O with an increased virulence and mating ability	1	>128	>128	>128	>128	>128
*C*. *neoformans *NR-41291	Isolated from human cerebrospinal fluid in China in July 2011.	1	>128	>128	>128	>128	>128
*C*. *neoformans* NR-41292	Isolated from human cerebrospinal fluid in China in February 2012.	1	>128	>128	>128	>128	>128
*C*. *neoformans* NR-41295	Isolated from human cerebrospinal fluid in China in February 2012.	1	>128	>128	>128	>128	>128
*C*. *gattii* NR-50185	Isolated from a goat in Aruba before the outbreak in Vancouver, British Columbia, Canada.	1	>128	>128	>128	>128	>128
*C*. *gattii* NR-50184	Isolated from a human during the outbreak in Vancouver, British Columbia, Canada.	1	>128	>128	>128	>128	>128
*C*. *gattii* NR-50423	Isolated from human cerebrospinal fluid in the Pacific Northwest region of North America.	1	>128	>128	>128	>128	>128
*C*. *gattii* NR-50422	Isolated from a human source (probably cerebrospinal fluid) in the Pacific Northwest region of North America.	1	>128	>128	>128	>128	>128
*C*. *gattii* NR-50186	A progeny of a genotypic cross between *C*. *gattii* strains R265 and CBS1930.	1	>128	>128	>128	>128	>128

^a^ Minimum inhibitory concentration (MIC), AmB (amphotericin B), RTV (ritonavir), LPV (lopinavir), ATV (atazanavir), SQV(saquinavir), NLF (nelfinavir).

### Screening PIs in combination with standard antifungals

Different PIs were evaluated in combination with AmB following CLSI guidelines [20]. Briefly, colonies from 48 hrs culture of *C*. *neoformans* H99 on YPD agar were resuspended in saline and diluted to 10^3^ CFU/mL in RPMI-1640 medium supplemented with a sub-inhibitory concentration of AmB (0.0625 μg/mL). Aliquots of 100 μL of the prepared inoculum were then dispensed in the wells of 96-well microtiter plates containing different PIs at a concentration of 16 μg/mL. Plates were incubated at 37°C for 72 hrs. The growth intensity of the individually treated cultures was measured spectrophotometrically at 540 nm and expressed relative to the growth of the untreated culture [21]. PIs reducing fungal growth by at least 80% were considered potential hits [15, 22].

### Microdilution checkerboard assay

The interaction between individual protease inhibitors and AmB was evaluated using a microdilution checkerboard assay as previously described [23–25]. Cryptococcal cells were adjusted to an inoculum of 10^3^ CFU/ml in RPMI-1640 medium and were treated with different concentrations of individual protease inhibitors (ranging from 1 to 32 μg/mL) combined with AmB (ranging from 0.015 to 4 μg/mL). The fractional inhibitory concentration index (ΣFICI) was used to describe the outcome of the combination as follows; synergy was defined at a value of ≤ 0.5, indifference values ranging from >0.5 to ≤4, and antagonism at values of >4 [26, 27].

### Time-kill assay

The growth kinetics of cryptococcal cells were evaluated as previously described [28, 29]. Briefly, yeast cells from overnight culture in YPD broth were diluted to ~10^4^ CFU/mL in RPMI-1640 medium. Cultures were treated individually with AmB (0.125 μg/mL), PIs (8 μg/mL), or their combination and incubated at 37°C. Aliquots from each culture were serially diluted at predetermined time points (0, 2, 4, 12, 24, and 48 hrs.) and plated on YPD agar incubated at 37°C for 48 hrs. The growth kinetics curves were set up by plotting the number of surviving cells versus time. A reduction in the fungal cell count  ≥ 3-Log10 compared to the control growth defines the fungicidal activity of the involved treatment compared to the untreated culture [30].

### Post-antifungal effect (PAFE) assay

The effect of PIs combination on the PAFE of AmB was performed as previously described [31, 32]. Briefly, an inoculum of 10^4^ CFU/mL of *C*. *neoformans* H99 in RPMI-1640 medium was treated with 1x MIC of AmB (0.25 μg/mL) alone or in combination with different PIs at a concentration of 8 μg/mL and incubated for 1 hr at 37°C. Treated fungal cells were collected by repeated cycles of centrifugation to remove the drug, then collected pellets were resuspended in RPMI-1640 and incubated at 37°C. Aliquots from different cultures were serially diluted and platted at different time points up to 24 hrs on YPD agar and incubated at 37°C for 48 hrs. PAFE was calculated using the following equation PAFE = T-C where T and C are the time required for cell population in treated and untreated cultures to increase by 1 Log10; respectively [33].

### Evaluation of the in vitro cytotoxicity on mammalian kidney cell line

The safety of the combination between AmB and PIs was evaluated on a monkey kidney epithelial cell line (Vero CCL-81) as previously described [34]. Briefly, Vero cells were seeded in flat bottom 96-well microtiter plate in an approximate density of 2x10^5^ cells/well in Eagle’s Minimum Essential Medium (EMEM) supplemented with 10% Fetal Bovine Serum (FBS) and 1% penicillin-streptomycin solution. Adherent cells were treated with AmB (1 μg/mL) alone or in combination with individual PIs (8 μg/mL) for 24 hrs at 37°C and 5% CO2. The viability of the treated cells was evaluated by incubating with a mixture of MTS (3-(4,5-dimethylthiazol-2-yl)-5-(3-carboxymethoxyphenyl)-2-(4-sulfophenyl)-2H-tetrazolium) and PMS (phenazine methosulfate) for 3 hrs. The absorbance of the formed formazan was measured at 490 nm [35].

### Evaluation of the in vivo efficacy of AmB/PIs combination in *Caenorhabditis elegans* model

The efficacy of the AmB/PIs was evaluated in the *C*. *elegans* nematode infection model as previously described [15, 36–38]. Briefly, nematode eggs of *C*. *elegans* AU37 (*Δglp-4; Δsek-1*) were harvested and hatched using an alkaline hypochlorite solution. The resultant progeny was transferred to *E*. *coli* OP50-cultured nematode growing medium (NGM) agar and incubated at 25°C for 72 hrs. Synchronized larvae in their L4 stage were collected and challenged with an inoculum of 10^6^ CFU/ml of *C*. *neoformans* H99 strain for 6 hrs. Infected larvae were incubated for 1 hr in M9 buffer containing 90 mg/liter kanamycin, 200 mg/liter streptomycin, and 200 mg/liter ampicillin, and repeatedly rinsed with saline to get rid of non-engulfed yeast and *E*. *coli* OP50 *cells*. Infected larvae were then treated with AmB (0.125 μg/mL) alone or in combination with individual PIs (8 μg/mL) in 20% RPMI-containing M9 buffer for 24 hrs. To evaluate the fungal burden, differently infected worms were vigorously vortexed using silicon-carbide beads. Then, the homogenates were plated on YPD agar and incubated at 37°C for 48 hrs.

### Statistical analysis

Statistical analysis was conducted using GraphPad Prism 8.0 Software (La Jolla, CA, USA). *P* values were calculated using one-way analysis of variance (ANOVA) with post hoc Dunnett’s test for multiple comparisons. *P* values of ≤ 0.05 were considered statistically significant.

## Results

### PIs enhance the killing efficacy of AmB

**Fig 2:**
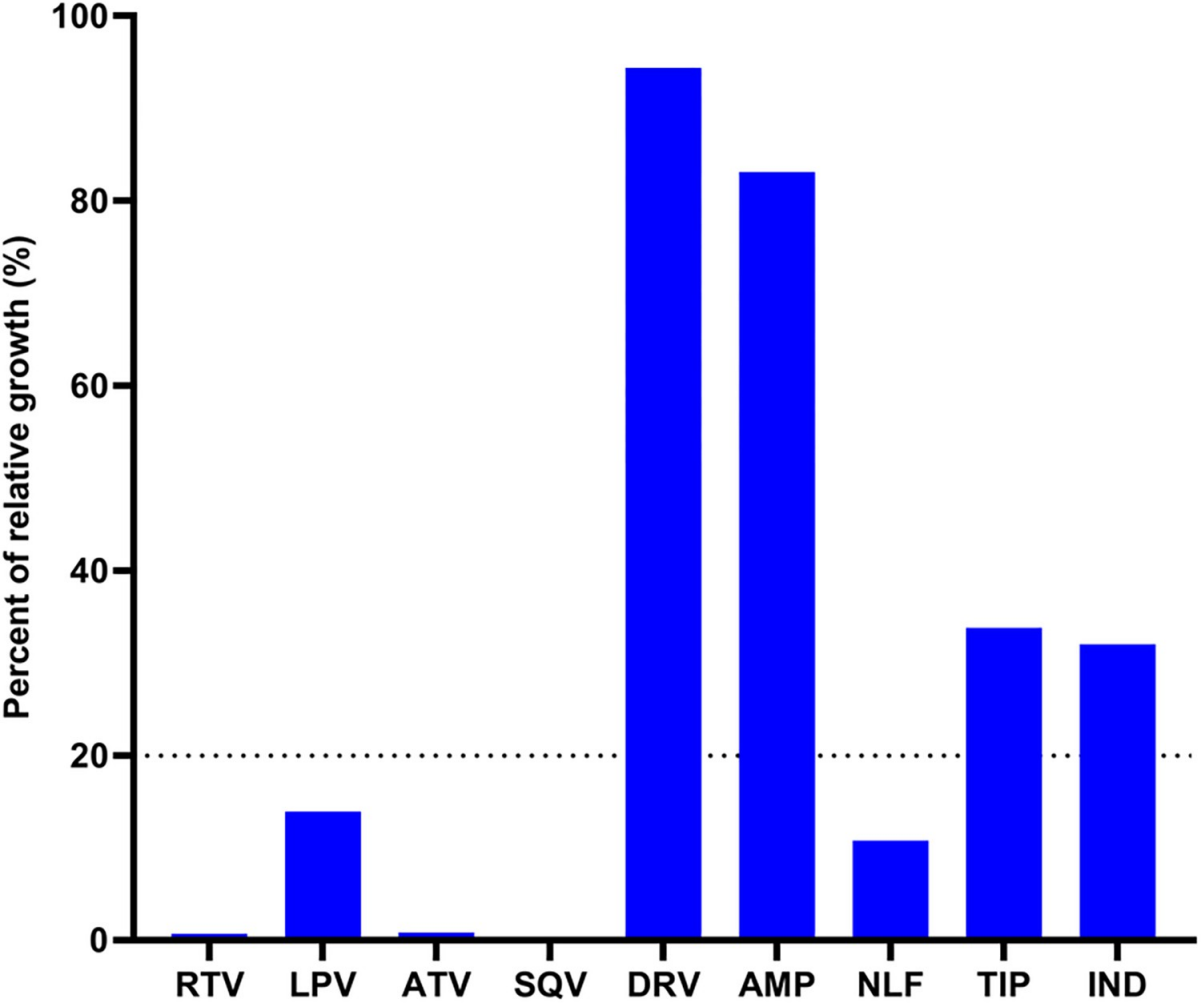
Initial screening of 9 FDA-approved protease inhibitors (PIs) in the presence of sub-inhibitory concentrations of amphotericin B (AmB). Cultures of ***C*. *neoformans*** H99 were individually treated with different PIs (16 μg/mL) in the presence of AmB (0.0625 μg/mL). The growth intensity of treated cultures was measured spectrophotometrically at 540 nm. Ritonavir (RTV), lopinavir (LPV), atazanavir (ATV), saquinavir (SQV), and nelfinavir (NLF) were identified as hit compounds reducing the growth of H99 below the cut-off value (20% relative to control growth) in the presence of AmB. Other PIs including amprenavir (AMP), indinavir (IND), darunavir (DRV), and tipranavir (TIP) were less effective.

Initially, we assessed the efficiency of nine clinically used PIs (ritonavir (RTV), lopinavir (LPV), atazanavir (ATV), saquinavir (SQV), nelfinavir (NLF), amprenavir (AMP), indinavir (IND), darunavir (DRV), and tipranavir (TIP)) as potential adjuvants of AmB. These drugs were screened at a fixed concentration (16 μg/mL) in the presence of a sublethal concentration of AmB (0.0625 μg/mL). Individual PIs inhibiting fungal growth by ≥ 80% were considered potential adjuvants of AmB. Five PIs demonstrated the ability to reduce the growth of *C*. *neoformans* H99 when combined with a sub-inhibitory concentration of AmB. Ritonavir (RTV), atazanavir (ATV), and saquinavir (SQV) completely suppressed the growth of the treated fungal cells. Similarly, lopinavir (LPV) and nelfinavir (NLF) showed a reduction in fungal growth by approximately 86% and 89%, respectively (Fig 2). On the other hand, amprenavir (AMP), indinavir (IND), darunavir (DRV), and tipranavir (TIP) demonstrate limited potentiating activity with AmB. Therefore, our focus shifted to evaluating the efficacy of the most active PIs (RTV, ATV, SQV, LPV, and NLF) in combination with AmB against clinical isolates of *C*. *neoformans* and *C*. *gattii*.

### PIs synergistically interact with AmB in microdilution checkerboard assay

The interaction between PIs and AmB was tested against a panel of isolates belonging to *C*. *neoformans* and *C*. *gattii* species using a standard microdilution checkerboard assay. Remarkably, as shown in Table 1, none of the PIs exerted any antifungal activity against all tested isolates, even at high concentrations (128 μg/mL). However, when combined with AmB, their synergistic effect reduced the minimum inhibitory concentration (MIC) of AmB up to 6 folds. The fractional inhibitory concentration indices (ƩFICI) were used to quantify the extent of the interaction, revealing that the five tested PIs exhibited synergistic interaction in combination with AmB against 100% of the tested strains, with ƩFICI values ranging between 0.09 and 0.5 (Table 2). It is worth noting that nelfinavir displayed an indifferent interaction with two of the tested isolates (Table 2).

**Table 2:** Microdilution checkerboard assay of amphotericin B (AmB) in combination with selected protease inhibitors (PIs).

Strain	AmB/RTV Combination			AmB/LPV combination			AmB/ATZ combination			AmB/SQV combination			AmB/NLF Combination		
	^a^MIC_C_ (μg/ml)	^b^ƩFICI	Mode	MIC_C_ (μg/ml)	ƩFICI	Mode	MIC_C_ (μg/ml)	ƩFICI	Mode	MIC_C_ (μg/ml)	ƩFICI	mode	MIC_C_ (μg/ml)	ƩFICI	Mode
*C*. *neoformans* NR-50332	0.0625/2	0.14	SYN	0.0625/2	0.14	SYN	0.0625/4	0.16	SYN	0.0625/1	0.13	SYN	0.25/2	0.51	IND
*C*. *neoformans* NR-50333	0.25/2	0.25	SYN	0.25/4	0.28	SYN	0.125/4	0.17	SYN	0.25/1	0.26	SYN	4/0.25	0.28	SYN
*C*. *neoformans* NR-50335	0.25/4	0.28	SYN	0.25/4	0.28	SYN	0.25/2	0.26	SYN	0.25/1	0.26	SYN	2/0.25	0.27	SYN
*C*. *neoformans* H99 ATCC-208821	0.0625/4	0.27	SYN	0.0625/4	0.28	SYN	0.0625/4	0.28	SYN	0.0625/2	0.27	SYN	0.0625/2	0.26	SYN
*C*. *neoformans* NR-48773	0.5/1	0.5	SYN	0.25/4	0.28	SYN	0.0625/8	0.13	SYN	0.25/2	0.27	SYN	2/0.25	0.27	SYN
*C*. *neoformans* NR-48768	0.25/8	0.31	SYN	0.25/4	0.28	SYN	0.125/4	0.17	SYN	0.25/1	0.28	SYN	2/0.25	0.27	SYN
*C*. *neoformans* NR-48774	0.125/2	0.14	SYN	0.125/4	0.16	SYN	0.125/8	0.19	SYN	0.0625/4	0.09	SYN	0.25/1	0.26	SYN
*C*. *neoformans* NR-48770	0.125/8	0.19	SYN	0.125/4	0.16	SYN	0.125/4	0.16	SYN	0.125/2	0.14	SYN	0.125/2	0.14	SYN
*C*. *neoformans* NR-48771	0.125/4	0.16	SYN	0.25/8	0.31	SYN	0.25/8	0.31	SYN	0.125/2	0.14	SYN	0.25/2	0.27	SYN
*C*. *neoformans* NR-48772	0.0625/16	0.18	SYN	0.125/8	0.19	SYN	0.125/4	0.16	SYN	0.0625/4	0.09	SYN	0.125/2	0.14	SYN
*C*. *neoformans* NR-48775	0.25/1	0.26	SYN	0.25/2	0.27	SYN	0.25/2	0.27	SYN	0.125/4	0.16	SYN	2/0.25	0.27	SYN
*C*. *neoformans* NR-48776	0.25/16	0.38	SYN	0.25/8	0.31	SYN	0.125/8	0.19	SYN	0.25/2	0.27	SYN	2/0.25	0.27	SYN
*C*. *neoformans* NR-41291	0.125/2	0.14	SYN	0.25/4	0.28	SYN	0.125/16	0.25	SYN	0.125/2	0.14	SYN	0.25/2	0.27	SYN
*C*. *neoformans* NR-41292	0.125/8	0.19	SYN	0.25/4	0.28	SYN	0.25/4	0.28	SYN	0.125/4	0.17	SYN	0.25/2	0.27	SYN
*C*. *neoformans* NR-41295	0.125/4	0.16	SYN	0.25/4	0.28	SYN	0.25/2	0.27	SYN	0.125/2	0.14	SYN	0.25/2	0.27	SYN
*C*. *gattii* NR-50185	0.25/8	0.31	SYN	0.25/4	0.28	SYN	0.0625/4	0.09	SYN	0.125/4	0.17	SYN	1/0.5	0.51	IND
C. gattii NR-50184	0.25/4	0.28	SYN	0.25/4	0.28	SYN	0.125/4	0.16	SYN	0.25/1	0.26	SYN	4/0.25	0.28	SYN
*C*. *gattii* NR-50423	0.25/2	0.27	SYN	0.25/2	0.27	SYN	0.25/2	0.27	SYN	0.25/1	0.26	SYN	2/0.25	0.27	SYN
*C*. *gattii* NR-50422	0.25/2	0.27	SYN	0.25/2	0.27	SYN	0.25/2	0.27	SYN	0.25/1	0.26	SYN	2/0.25	0.27	SYN
*C*. *gattii* NR-50186	0.25/4	0.28	SYN	0.25/4	0.28	SYN	0.0625/8	0.13	SYN	0.25/1	0.26	SYN	2/0.25	0.27	SYN

^a^ Minimum inhibitory concentration of amphotericin B and corresponding HIV protease inhibitor in combination.

^b^ Fractional inhibitory concentration index (ΣFICI) was used to define the interactions between AmB and individual PIs based on the following values: synergy (SYN) at ΣFICI values of ≤0.5, indifference (IND) at ΣFICI values ranging from >0.5 to ≤4, and antagonism at ΣFICI values of >4. Tested PIs include ritonavir (RTV), lopinavir (LPV), atazanavir (ATV), saquinavir (SQV) and nelfinavir (NLF).

### PIs impact the growth kinetic of cryptococcal cells when combined with AmB

**Fig 3:**
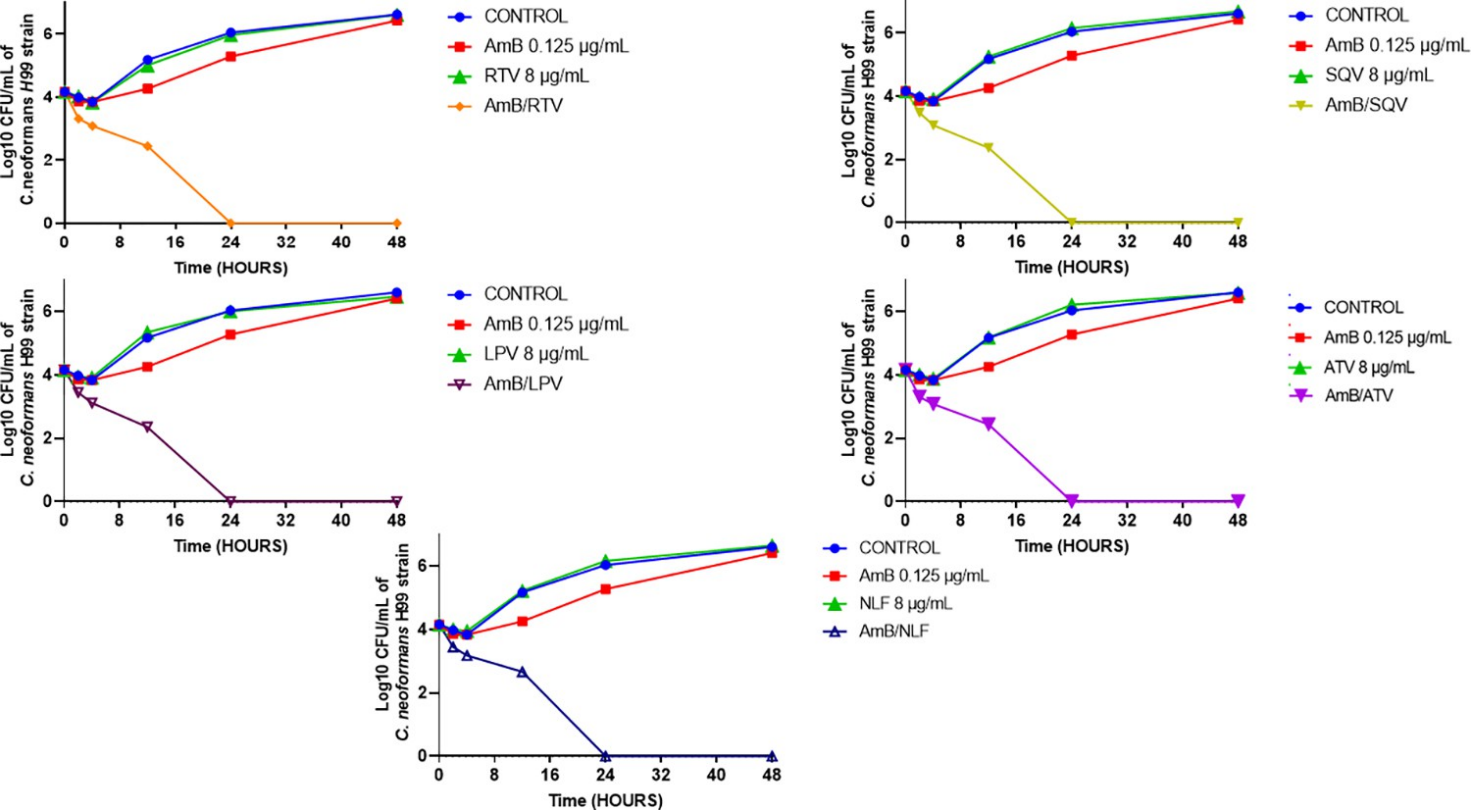
Growth kinetics of *C*. *neoformans* H99 treated with protease inhibitors (PIs) in combination with amphotericin B (AmB). Cultures of *C*. *neoformans* H99 were individually treated with PIs; including ritonavir (RTV), lopinavir (LPV), atazanavir (ATV), saquinavir (SQV) and nelfinavir (NLF) at 8 μg/mL; alone or in combination with AmB (0.125 μg/mL). The growth of treated cultures was evaluated for 48 hrs. The fungicidal activity was defined as ≥ 3 log10 CFU/mL reduction compared to the untreated control (DMSO).

Next, we assessed the effect of PIs on the killing activity of AmB using a time-kill assay. As shown in Fig 3, neither individual PIs (8 μg/mL) nor AmB (0.125 μg/mL) could independently exert any effect on the proliferation of the cryptococcal cells. However, their combination exhibited a fungicidal effect, completely eradicating the treated population within 24 hours.

### PIs extend the post-antifungal effect (PAFE) of AmB

To evaluate the impact of combining HIV-protease inhibitors on the PAFE of AmB, an inoculum of 10^4^ CFU/mL was exposed to AmB at 1x MIC (0.25 μg/mL) in the presence of a fixed concentration of PIs (8 μg/mL). AmB showed an average PAFE of 4 hours. However, when combined with PIs, AmB showed an extended effect on the proliferation capability of the growing yeast cells, with an average PAEF of 10 hours (Table 3).

**Table 3:** The effect of protease inhibitors on PAFE of amphotericin B on *C*. *neoformans* H99.

Treatment	PAFE (hrs)
AmB (0.25 μg/mL)	4
AmB/RTV	10
AmB/LPV	10
AmB/ATV	10
AmB/SQV	10
AmB/NLF	10

### AmB/ PIs combination does not induce additional toxicity to kidney epithelial cells

**Fig 4:**
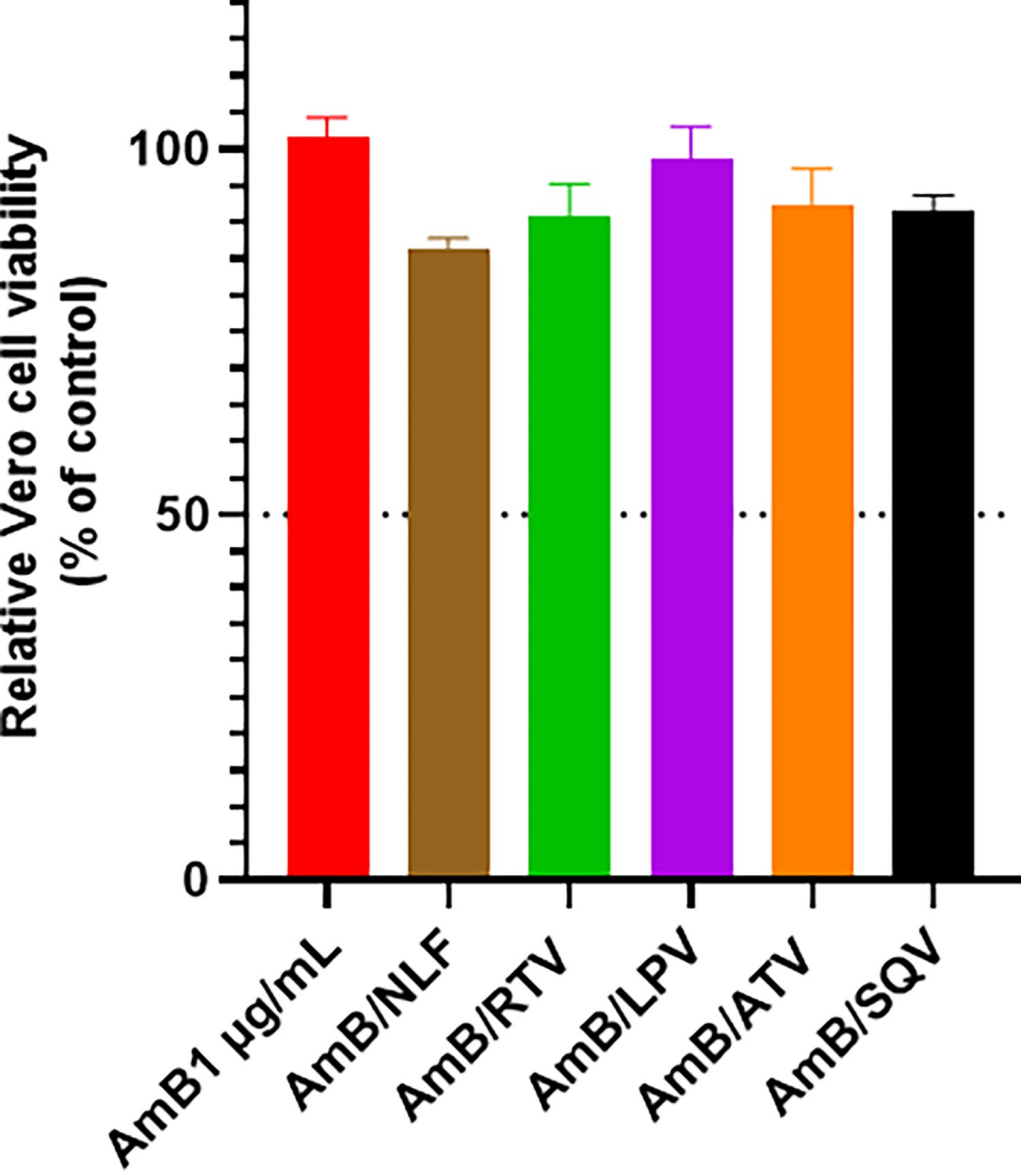
Cytotoxicity assay of amphotericin B (AmB) and protease inhibitors (PIs) on a mammalian kidney cell line. Vero cells were treated with AmB at 1 μg/mL, representing MIC_90_ of AmB, alone or combined with different PIs (8 μg/mL). The viability of the treated cells was evaluated after 24 hrs using MTS/PMS colorimetric assay. The absorbance was measured at 490 nm and the viability was expressed relative to untreated culture.

The potential of PIs to exacerbate renal toxicity of AmB was assessed by examining their combinatory in vitro effect on Vero cells using MTS/PMS colorimetric assay. As shown in Fig 4, AmB at its MIC_90_ (1 μg/mL) did not impact the viability of the treated cells. Likewise, when combined with PIs, the treatment was non-toxic, with no statistical difference in the viability of various treated cultures.

### PIs enhance the killing activity of AmB in vivo using the *Caenorhabditis elegans* nematode model of cryptococcosis

**Fig 5:**
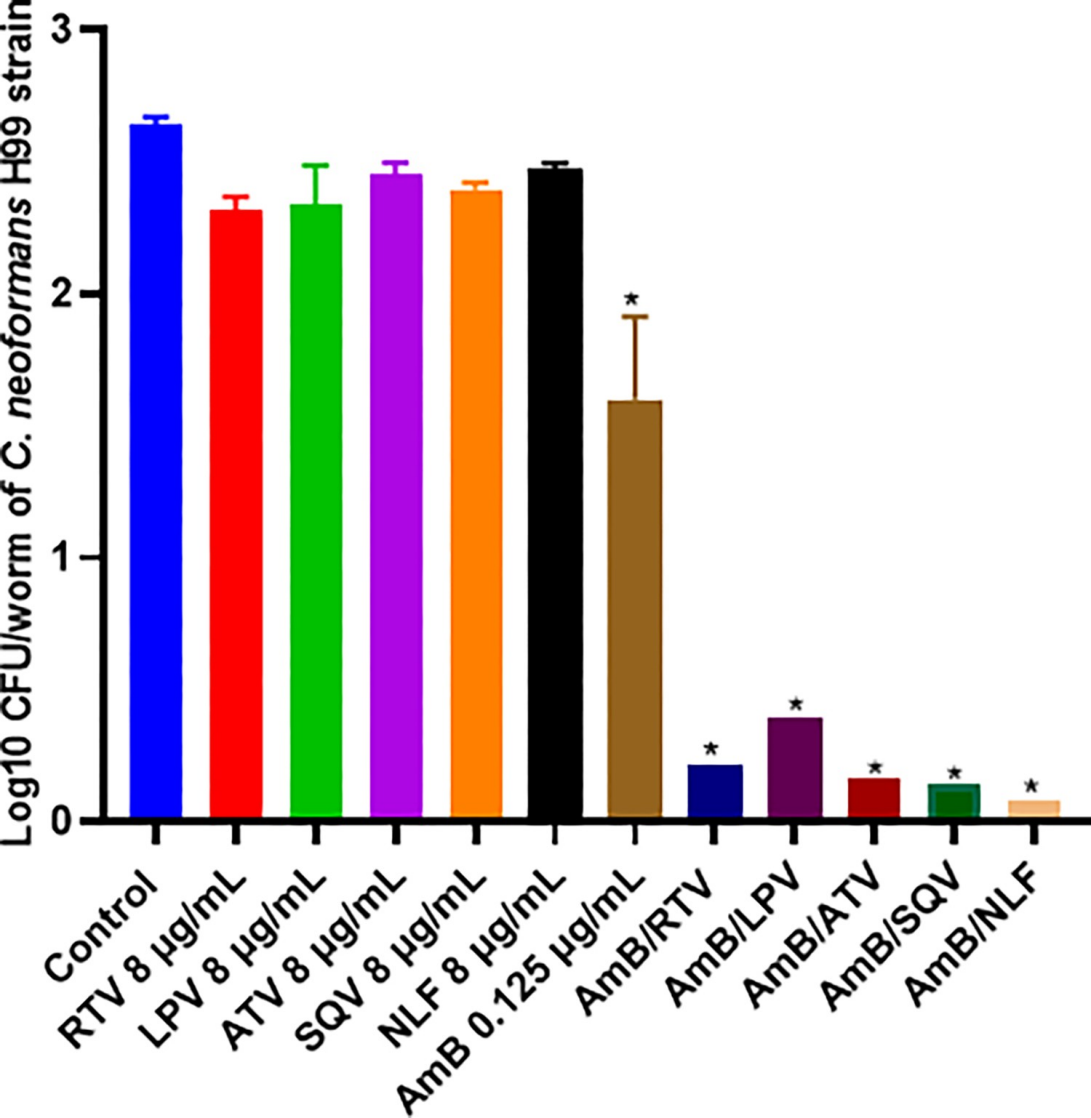
In vivo efficacy of amphotericin B (AmB) combination with protease inhibitors (PIs) in a *C*. *elegans* nematode model of cryptococcosis. L4-stage larvae infected with *C*. *neoformans* H99 strain were treated with AmB alone (0.125 μg/mL) or combined with PIs (8 μg/mL). Untreated worms were involved as a negative control. The fungal burden of treated worms was evaluated 24 hrs following the treatment. Asterisks indicate a statistical significance (*P* value < 0.05, as determined by one-way analysis of variance [ANOVA] using Dunnett’s test for multiple comparisons) compared to the untreated control.

We exploited the nematode model of *C*. *elegans* to assess whether the enhanced killing activity of PIs, in combination with AmB, could be translated in vivo. Synchronized L4-larva of *C*. *elegans* infected with *C*. *neoformans* H99 strain were treated with PIs (8 μg/mL), AmB (0.125 μg/mL), or a combination of PIs/AmB at the same concentrations. As shown in Fig 5, PIs alone did not exert a significant effect on the fungal load of treated nematodes (2.39 ± 0.06 Log10 CFU/worm) compared to the control group (2.64 ± 0.029 Log10 CFU/worm). In contrast, the AmB treatment alone resulted in a significant reduction in the fungal load of the treated nematodes (~1.04 Log10 CFU/worm). Furthermore, combined with PIs, it exhibited enhanced resolving activity with a mean reduction of ~2.44 and 1.40 Log10 CFU/worm compared to the control group and AmB-treated group, respectively.

## Discussion

Cryptococcosis is an opportunistic fungal infection caused mainly via the inhalation of viable yeast cells or spores of *C*. *neoformans*. While individuals with healthy immune systems typically neutralize these invading cells, immunocompromised patients usually exhibit morbidities ranging from mild pulmonary infection to life-threatening meningitis [39]. The continuous global growth of the immunocompromised population as well as the increased usage of immunosuppressant therapies, amplifies the risk of cryptococcal infection among this diverse group of patients. One study indicated that 27% of cryptococcosis patients were HIV-positive [39]. Furthermore, the scarcity of proper treatment regimens, especially in low-income countries with the highest share of the HIV population, directly contributes to the increased global burden of this infection. In 2022, UNAIDS estimated that 39 million are currently living with HIV worldwide, with 53.3% of this estimate (20.8 million) residing in Eastern and Southern Africa, where 500,000 new cases are reported annually [40].

The current regimen requires continual optimization due to host toxicity and pathogen resistance in addition to the scarcity of its components [8, 41]. AmB is the primary antifungal used for the induction regimen of cryptococcosis treatment. Deoxycholate AmB (0.7–1 mg/kg/day) is given through intravenous infusion for 2 weeks to provoke quick and efficient CSF sterilization. However, AmB-associated toxicity favors its combination with 5-FC (100 mg/kg/day) to mitigate toxicity and enhance early-killing activity [42, 43]. Limited availability of 5-FC, primarily due to cost constraints, poses challenges to treatment accessibility, particularly in high-burden regions [44]. On the other hand, fluconazole is mainly used in moderate to low doses for both consolidation and maintenance stages of the treatment due to its fungistatic activity. Its wide availability in regions with limited access to AmB and 5-FC favors its use as an alternative therapy leading to a higher rate of treatment failure and relapses [45, 46]. The identification of cost-effective drugs like PIs that synergize with AmB, enhancing its efficacy, could reduce required doses, associated toxicity, and overall treatment costs.

In this study, we evaluated nine FDA-approved PIs as potential adjuvants to AmB for cryptococcosis treatment. Five PIs (ritonavir (RTV), lopinavir (LPV), atazanavir (ATV), saquinavir (SQV), and nelfinavir (NLF)) suppressed the fungal growth of the *C*. *neoformans* H99 strain by more than 80% relative to control growth in the presence of a sublethal level of AmB. This synergistic activity was further confirmed by evaluating the extent of this interaction using a microdilution checkerboard assay, as well as its effect on growth kinetics using a time-kill assay. Here, these individual PIs interacted synergistically with AmB against all tested isolates, resulting in an average 6-fold reduction in the MIC. In the time-kill assay, PIs exhibited fungicidal activity in combination with a sublethal concentration of AmB, eradicating the entire cryptococcal population within 24 hrs. This promising activity could allow for the usage of a lower dose of AmB without compromising the overall killing efficacy required for efficient CSF sterilization, similar to the combination of AMB and 5-FC [47]. Unlike 5-FC, against which *C*. *neoformans* rapidly develop resistance at high frequency, PIs possess no individual antifungal activity against *C*. *neoformans*, therefore reducing the likelihood of resistance development [48].

Next, we evaluated the potential impact of PIs on the PAFE of AmB. Like the time-kill assay, PAFE is a critical parameter that influences the dosing interval of used antimycotics. While the time-kill assay evaluates how continuous exposure to increasing concentrations of the antifungals affects the growth of treated cells, PAFE measures the duration of growth inhibition after brief exposure to antifungal agents, simulating what occurs in vivo when the antifungal concentration falls below its MIC due to pharmacokinetic parameters [49, 50]. Remarkably, our findings revealed that *C*. *neoformans* H99 inoculum exposed to AmB at its MIC exhibited an approximate delay of 4 hrs to increase by 1 Log10, which is consistent with a previous study [51]. On the other hand, AmB/PIs exhibited an extended PAFE for an additional 6 hrs which may allow the application of a less frequent AmB regimen once this combination is evaluated in a clinical setting.

Nephrotoxicity is the main adverse effect observed among patients receiving AmB-based treatment regimens. A previous study reported acute kidney injury in 60.8% of patients receiving AmB [52]. This toxicity can be exacerbated by factors such as mild renal insufficiency, certain infections like HIV, or due to concurrent use of certain drugs including steroids, immunosuppressants, and certain antibiotics (such as vancomycin and imipenem) [53–55]. Therefore, we investigated whether co-administration of different PIs would impact the cytotoxicity of AmB using the Vero cell line. Consistent with previous studies, AmB at its most frequent MIC against *Cryptococcus* isolates (1 μg/mL) did not exhibit any toxicity on treated Vero cells [56, 57]. Similarly, adding PIs at the tested concentration did not affect the safety profile of AmB, indicating the safety of this combination on renal tissue.

Finally, we evaluated the in vivo efficiency of PIs/AmB combination in the cryptococcosis *C*. *elegans* nematode model. *C*. *elegans* has emerged as a valuable tool in studies involving clinically relevant fungi, including *Cryptococcus spp*., aiding in the identification and study of various virulence factors and antifungal agents [58, 59]. If left untreated, *C*. *neoformans* is typically lethal to *C*. *elegans*, with factors such as polysaccharide capsule and specific genes known for mammalian virulence also influencing nematode mortality [60]. Research utilizing *C*. *elegans* has highlighted the effectiveness of drug combinations with antifungals in treating infected nematodes in vivo [61–63]. The PIs practically eradicated the fungal burden of the infected worms in combination with a sublethal dose of AmB, achieving approximately 2.44 Log10 reduction (92.4%) compared to the burden of untreated worms. AmB alone was able to only reduce fungal burden by 1.04 Log10 reduction (39.4%) compared to untreated worms. As expected, PIs alone did not have any significant reduction in fungal burden. This observation suggests a potential clinical significance of PIs as an adjunct therapy for treating cryptococcal infections, especially in HIV patients where PIs are already commonly prescribed.

In conclusion, the limited cost of PIs and their availability in limited-resource areas compared to other novel antifungal agents make them an attractive option for combination therapy, especially in regions with a high disease burden and limited access to more expensive drugs such as flucytosine.

## Supporting information

S1 Data (XLSX)
